# Exploring stakeholder perspectives on nursing competencies in palliative care in India: a qualitative inquiry

**DOI:** 10.1186/s12904-026-01998-1

**Published:** 2026-02-05

**Authors:** Soumya Liz Jacob, Malathi G Nayak, Linu Sara George, Judith Angelitta Noronha, Baby S Nayak, Shashidhara Y N, Leah Macaden, Anuja Dwarkadas Damani, Vani Lakshmi R, Suba Sooria Panchalingam, Sangeetha N Murugan

**Affiliations:** 1 Department of Community Health Nursing, Manipal College of Nursing, Manipal Academy of Higher Education , Karnataka Manipal, 576104 India; 2 Department of Fundamentals of Nursing, Manipal College of Nursing, Manipal Academy of Higher Education, Manipal, Karnataka 576104 India; 3 Department of Obstetrics & Gynaecological Nursing, Manipal College of Nursing, Manipal Academy of Higher Education, Manipal, Karnataka 576104 India; 4 Department of Child Health Nursing, Manipal College of Nursing, Manipal Academy of Higher Education, Karnataka 576104 Manipal, India; 5 School of Health in Social Science, The University of Edinburgh, Edinburgh, Scotland EH8 9AG United Kingdom; 6 Department of Palliative Medicine and Supportive Care, Kasturba Medical College, Manipal Academy of Higher Education, Manipal, Karnataka 576104 India; 7 Department of Health Technology and Informatics, Prasanna School of Public Health, Manipal Academy of Higher Education (MAHE), Manipal, Karnataka 576104 India; 8 Department of Nursing Service, Kasturba Hospital, Manipal Academy of Higher Education , Manipal, 576104 India; 9 Department of Education and Research, Bangalore Hospice Trust – Karunashraya Marathahalli, Bangalore, 560 037 India

**Keywords:** Nursing competencies, Palliative care, Stakeholder perspectives, Qualitative, India

## Abstract

**Background:**

India faces a growing need for Palliative Care due to its ageing population, rising cancer burden, and high prevalence of chronic illnesses. Unfortunately, less than 4% of the population has access to Palliative services, and the country ranks 59th in the 2021 Quality of Death Index. Contextually relevant and culturally sensitive nursing competencies are crucial to address this gap. However, there is no structured, evidence-based palliative nursing competency framework tailored to India’s sociocultural and healthcare realities. This study explored stakeholder perspectives to identify core nursing competencies required for palliative care in the Indian context.

**Methods:**

A qualitative design was employed, comprising seven Focus Group Discussions with nurses involved in delivering care to patients with life-limiting illnesses, and thirty five In-Depth Interviews with patients and caregivers at various stages of the disease trajectory, from diagnosis to terminal illness and end-of-life. Data were analysed using Braun and Clarke’s thematic analysis approach. Line-by-line coding was conducted using Open Code 4.02 version to systematically identify themes and subthemes. These were synthesized into competency domains and statements that reflect the essential skills, knowledge, and attitudes required for palliative nursing in India. To ensure the rigor and trustworthiness of the findings, measures such as member checking and peer debriefing were undertaken throughout the research process.

**Results:**

Thematic analysis yielded seven distinct competency domains. These domains captured a wide range of nursing roles, including understanding foundations of Palliative Care, communication, ethical, legal, and professional responsibilities, symptom management and enhancing comfort, psychosocial, cultural, and spiritual aspects of care, and team collaboration. The findings emphasized the importance of culturally grounded, holistic, and compassionate care tailored to the needs of Indian patients and their families. Importantly, region-specific factors, such as Indian family dynamics, cultural attitudes towards death, and spiritual beliefs, emerged prominently during the thematic analysis. Interestingly, there was a notable convergence between the views expressed by nurses in focus group discussions and those of participants in in-depth interviews regarding essential palliative care competencies.

**Conclusions:**

This study presents an empirically derived set of thematic domains and insights for palliative care nursing competencies, grounded in stakeholder perspectives and tailored to the Indian context. The identified domains can inform curriculum development, training programs, and policy formulation to strengthen palliative care services across India.

**Trial registration:**

The study is registered in the Clinical Trials Registry of India CTRI/2023/07/055216) dated 14/07/2023.

**Supplementary Information:**

The online version contains supplementary material available at 10.1186/s12904-026-01998-1.

## Background

As the world’s population ages and the prevalence of cancer and other chronic diseases rises, there is an increasing need for palliative care [1]. Palliative care (PC), recognized as a fundamental human right [2], offers multidimensional assistance to patients with life-limiting illnesses and their families in an effort to alleviate significant health-related suffering [3]. While 76% of the need is found in low- and middle-income countries (LMICs), PC is still scarce or non-existent in the majority of these nations [1]. The World Health Assembly emphasized in 2014 that all healthcare practitioners have an ethical obligation to provide palliative care as part of their national health systems [4].

India, being the most populous country in the world and a land of diversity and social and economic inequalities, has an estimated prevalence of PC needs of 1.5 to 43.1/1000 population [5]. The Quality of Death Index (2021) ranks India in the 59th position out of 80 countries [6]. Although there have been advances in the recent past, less than 4% of the Indian population has access to PC [7]. India is home to a diverse array of religious and cultural customs, each with its unique belief systems and perspectives on death and illness [8]. In Hinduism, suffering is often viewed as a consequence of past deeds, and the balance of good and bad deeds is believed to influence rebirth. Death is accepted and often approached through detachment, prayer, and atonement, with many preferring to die at home surrounded by family. Buddhism also emphasizes reincarnation and detachment, discouraging opioids at life’s end. Christianity links suffering to sin, with confession believed to absolve sins, while Islam sees suffering as submission to God and permits pain relief in terminal patients. All major religions oppose suicide and euthanasia [9]. Although religion significantly influences end-of-life decisions, multiple nonreligious factors also impact patients’ attitudes and preferences regarding death and dying rituals [10]. Thus, it is essential to have an effectively integrated PC service that is culturally and socially appropriate.

This study aimed to explore and understand the perspectives of nurses, patients, and caregivers regarding the nursing competencies necessary to deliver palliative care. It forms part of a larger research initiative aimed at developing a contextually appropriate PC nursing competency framework across various healthcare settings within the Indian context.

## Objectives

To describe the perceptions and experiences of patients with life-limiting illness regarding nursing competencies for PC in India.

To understand the views and expectations of family caregivers on the knowledge, skills, and attitudes required by nurses to provide PC.

To identify and describe the PC nursing competencies that nurses consider to be critical while caring for patients with life-limiting illnesses.

### Theoretical assumptions

This study is grounded in a constructivist epistemology, recognizing that knowledge is co-constructed through social interactions and individual experiences [11] thereby allowing both the researcher and participant to influence the data on the basis of their experiences and background [12]. A scoping review on nursing competencies in PC was conducted by the researcher as the primary investigator, which guided her at various steps of this qualitative research. Anchored in an experiential orientation [13], the research prioritized the subjective meanings and lived realities of patients, caregivers, and nurses associated with life-limiting illness. Through In-Depth Interviews (IDIs) with patients and Focus Group Discussions (FGDs) with nurses, the study aimed to uncover the understandings of PC competencies shaped by context, relational dynamics, and practice. The research adopted a predominantly inductive approach, allowing insights to emerge from the data rather than being imposed by preexisting theories [14]. Thematic analysis incorporated both semantic (surface-level) and latent (underlying) coding to capture explicit expressions as well as deeper, implicit meanings [13]. The interviews and focus groups were conducted by the primary author, a female registered nurse with a Master of Science in Nursing and formal training in qualitative research. Prior to commencing her PhD studies, she was actively engaged in clinical practice in long-term care, with a focus on caring for individuals with life-limiting conditions. Her background in clinical nursing and qualitative research equipped her to sensitively engage participants and thoughtfully interpret the data within the context of palliative care.

## Methods

A descriptive qualitative design [15] was employed, involving in-depth interviews with patients and caregivers, as well as focus group discussions with frontline nurses. This design was used to provide a rich, contextualized description of palliative care nursing competencies as experienced and interpreted by patients, family caregivers, and nurses.

### Setting and participants

The study sites included a tertiary reference hospital and a PC centre in southern India.

Participants were recruited via purposive sampling with maximum variation across multiple medical specialties, including neurology, nephrology, cardiology, oncology, respiratory medicine, and palliative care, to ensure a heterogeneous representation of their clinical experiences and patient demographics. A total of 35 one-to-one IDIs (In-Depth Interviews), comprising 20 patients and 15 caregivers, were conducted. The eligibility criteria for patient participants included a confirmed diagnosis of a terminal condition and the ability to meaningfully participate in the interview process. Patients were excluded if they demonstrated impaired coherence, were experiencing hemodynamic instability due to advanced illness, or declined to provide informed consent. In addition, primary family caregivers of terminally ill patients who were emotionally stable and consented to participate were also included in the study to provide their experiences and perspectives on the nursing competencies of PCs.

Seven FGDs (Focus Group Discussions) were conducted with registered nurses, each comprising between six and eight participants [16], with a total of 50 nurses taking part. Participants were purposively selected to capture a range of clinical expertise and professional backgrounds. The inclusion criteria for nurse participants included full-time employment at the selected healthcare institutions, a minimum of one year of continuous post-qualification clinical practice, and direct patient care provision to those with life-limiting conditions. In this study, nurses working in tertiary reference centres were referred to as generalists, whereas those working in palliative care centres were referred to as specialists.

### Data collection

Data collection took place from November 2023 to May 2024. The interview topic guide used for both FGDs and IDIs was developed by the primary researcher, based on an extensive review of relevant literature and a contextual understanding of the study setting to ensure that the questions were both theoretically grounded and contextually appropriate. To assess content validity, the topic guide was reviewed by a panel of seven experts in the fields of PC, nursing education, and social work with qualitative research experience, all of whom held either a PhD or MD qualification. Each expert was asked to evaluate the relevance and clarity of each item in the guides via a four-point scale (1 = not relevant, 2 = somewhat relevant, 3 = quite relevant, 4 = highly relevant) [17]. The Item-Level Content Validity Index, I-CVI, was 0.80, which was considered acceptable for each item [18]. This indicated that the topic guide possessed a satisfactory level of content validity for the intended qualitative inquiry. Minor revisions were made to the guide in response to expert feedback. These included improvements in sentence construction for clarity and the addition of “place of residence” to the sociodemographic section to enhance contextual relevance in participant profiling, which was finally approved by the co-authors.

**Table:** 

Focus Group Discussion Guide (Nurses)
1. Can you describe your understanding of Palliative care?
2. What do you think are the roles of nurses in Palliative Care?
3. What do patients and relatives value the most while you render them care?
4. Can you come up with the various problems faced by patients with life-limiting illnesses?
• What are the common physical problems faced by patients with life-limiting conditions?
• What are the common psychological/emotional problems faced by patients with life-limiting illnesses?
• What are the other practical problems faced while caring for such patients?
5. What are the other competencies you need to possess to effectively take care of patients with life-limiting conditions during the various phases of their illness?
6. How do patients and relatives usually thank you for your work?
7. Are there any other recommendations from your experience to render effective palliative care?

**Table:** 

In-depth interview guide for Patients Caregivers
1. Can you think of interactions with nurses that made you feel good during your disease course/hospital stay? Can you explain more about it? Why was it so special?
2. Can you think of situations where you felt that the care you received was not up to your expectations? What do you think could have been better done?
3. What are the skills/qualities you think nurses should possess while caring for patients like you/your loved one?
4. What kind of help may you require from nurses in the future?
5. Spending time with you was very valuable. Do you have any other thoughts or reflections regarding the topic we discussed?

There was no prior relationship between the researcher (primary author) and the participants before the commencement of the study. Selected participants were approached individually, and the researcher formally introduced herself prior to data collection. She explained the purpose of the study, the voluntary nature of participation, participants’ right to withdraw at any point, and the assurance of anonymity and confidentiality. These details were also included in the Participant Information Sheet, which was provided in print to each participant before obtaining written informed consent.

After informed consent (written), FGDs and IDIs- were conducted within the premises of the designated study settings with adequate privacy. The durations of FGDs and IDIs ranged from 54 to 93 min [19] and 29–42 min [20], respectively. Most FGDs were conducted in English. However, in two instances, some participants preferred to express themselves in their native languages—Kannada and Malayalam. The FGD moderator and the primary researcher were fluent in at least one of these languages, facilitating effective communication during the sessions. Patient interviews were conducted in Kannada, Konkani, English, Hindi, Telugu, and Malayalam. Trained translators proficient in each language were present to ensure accurate translation and interpretation during the interviews [21]. The interviews were conducted until data saturation was reached [22]. Each FGD and IDI was audio recorded via an audio recording device. Field notes were documented to understand contextual realities [23]. COREQ guidelines for reporting qualitative research were followed [24] (Supplementary material 1).

Data were anonymized and transcribed within 24 h of collection by the primary researcher and translator as needed. The primary researcher listened to the interviews several times and compared them with the field notes during transcription. The services of translators were utilized when transcribing 15 Kannada, 1 Konkani, and 1 Telugu IDI, as the primary researcher was not proficient in these languages. Part of one FGD was also transcribed with the help of a translator, as it was conducted in the Kannada language. Once transcribed to English, these transcriptions were retranslated and peer reviewed by academic colleagues for rigor and trustworthiness [25]. The transcripts were then exported into the qualitative analysis software- Open Code version 4.02 [26]. Data from each category (generalist and specialist nurses in the FGD and patients and caregivers in the IDI) were coded separately.

The data were analysed using the six-phase approach to thematic analysis outlined by Braun and Clarke [27]. An interpretive and inductive approach using an iterative thematic analysis was conducted, guided by constant comparison [28]. The process of repeatedly listening to the audio recordings, transcribing the content verbatim into a Word document, and conducting multiple readings of the transcripts, enabled the researcher to immerse in the qualitative data. This allowed the identification of meaningful text segments, followed by text reduction to filter relevant content, producing initial codes. These codes were then categorized into thematic domains based on conceptual similarities after being improved for consistency and clarity as shown in Table 1.

**Table 1:** Overview of Thematic Analysis

**Steps of thematic analysis**	**Actions undertaken**
Familiarization with the data	All transcripts were read multiple times to familiarize the data Integration with the field notes to better understand the contextual depth.
Generating initial codes	Initial codes were generated systematically through line-by-line coding of the transcripts, capturing key features of the data relevant to the research question. Codes were created inductively, staying close to the participants' language and reflecting their perceptions and experiences with palliative nursing. Relevant memos were attached to the codes to capture analytical thoughts during the coding process.
Combining codes into themes.	Codes were grouped into potential themes by identifying patterns and relationships within the data. Cross-coding was carried out to ensure methodological rigor.
Reviewing themes.	The fourth phase involved reviewing and refining these themes to ensure they accurately represented the coded data and the dataset as a whole. Refinement of themes occurred by combining, splitting, or discarding elements based on their depth, relevance, and recurrence in the data.
Determine the significance of themes.	In the fifth phase, themes were mapped to domains with relevant illustrative quotes to support each theme.
Reporting of findings	A detailed account of the themes was produced, integrating insights from field notes to provide contextual depth. All authors independently reviewed the themes and transcripts, and through collaborative discussion, reached consensus on the final thematic structure.

Table 2 illustrates a sample of the iterative process used to generate themes from IDI data. While some themes emerged as distinct and standalone, others were combined through the refinement process.

**Table 2:** Inductive coding procedure for Patient Centered Care

Reduced Verbatim	Code	Subtheme	Theme
*“They are taking care of everything.”(IDI-P3)*	Taking care	Comprehensive support	Patient Centred Care
*“They take me for a walk, and we watch programs.”(IDI-P3)*	Walking, watch a program	Engagement beyond routine	
*“Here, they find what I want and do it.”(IDI-P2)*	Find out	Proactive attention	
*“They are just treating me as their own grandfather—that is enough.”(IDI-P9)*	Treat as their own	Personalized care	
*“They should understand and do what is needed without us asking daily.”(IDI-P7)*	Do what is needed	Intuitive understanding	
*“If I live or die, it should be at home.”(IDI-P3)*	Die at home	Respect for patient wishes	
*“I am dependent on them for everything—even going to the washroom.”(IDI-P2)*	Dependent	Support for daily needs	

### Trustworthiness of the study

To ensure the rigor and trustworthiness of this study, the four criteria proposed by Lincoln and Guba (1985)—credibility, transferability, dependability, and confirmability—were employed throughout the study [29].

Credibility was established through the use of the triangulation method, which combined data from FGDs and IDIs with patients and caregivers, respectively [30]. This allowed a comprehensive understanding of the phenomenon from multiple perspectives. In order to ensure accurate representation, member checking involved summarizing important topics from conversations and interviews and verifying them with participants [31]. To lessen bias and improve interpretive accuracy, the coding procedure and emerging themes were further reviewed through peer debriefing [25] with academic colleagues. The shortened text, initial coding, and thematic groups were examined by three peers with qualitative experience who were not involved in the data collection process. Feedback from co-authors was used to challenge assumptions, ensure consistency in the code application, and confirm the coherence of domain classifications.

In-depth explanations of the study environment, participant traits, and contextual elements are given here to promote transferability [32]. The diversity of samples enhanced the relevance of the findings to similar healthcare settings. Sufficient contextual detail is provided to enable readers to assess the applicability to their own settings. An audit trail documenting decisions related to data collection, coding, and thematic analysis was maintained throughout the study [25], including records of interview guide development, data management, and code refinement. A code–recode strategy was employed to verify consistency over time, and dependability was further strengthened through the review of the coding process by academic colleagues and co-authors experienced in qualitative research.

Confirmability was addressed through reflexive journaling, in which the researcher documented personal reflections, potential biases, and analytic decisions throughout the research process [23]. The use of verbatim quotes from participants in the findings section demonstrates that the results are grounded in participants’ experiences rather than researcher interpretations.

The iterative thematic analysis revealed strong convergence across the two datasets. Most of the competency areas that nurses recognized were also reported by patients and caregivers, indicating a common understanding of palliative nursing competencies and supporting the validity of the resulting framework. Financial burden and the rural-urban divide surfaced in the IDIs, whereas advance directive implementation, differences in end-of-life practices between centres, boundaries in nurses’ professional scope, and sociocultural factors such as nondisclosure of diagnosis and the degree of family involvement were only discussed in the FGDs. The active engagement of nurses, patients, and caregivers was in line with codesign principles [33]. This aided the development of the framework, ensuring that the skills are grounded in real-world experience and tailored to the regional context.

### Ethical considerations

Ethical approval for the study was obtained from the institutional ethics committees of the respective centres where the research was conducted (IEC approval reference numbers: IEC1 59/2023 and EC/NEW/INST/2024/KA/0398). Additionally, separate permissions were obtained from the heads of the departments from which participants were recruited. Written informed consent was obtained from all participants prior to their enrolment in the study. The participants were provided with an information sheet detailing the study’s objectives, the associated questionnaire, voluntary participation, the procedures involved, and assurances of anonymity and confidentiality.

## Results

A total of 50 nurses participated in the FGDs. Of these, 28 were classified as generalists working in areas such as Oncology, Neurology, Medicine, Intensive Care Units (ICU), and Outpatient Departments. The remaining 22 were classified as specialists working in dedicated palliative care units. Additionally, in-depth interviews were conducted with 20 patients and 15 caregivers. Table 3 describes the characteristics of the FGD participants.

Table 3 presents the sociodemographic and professional characteristics of nurses who participated in the FGDs. All specialist nurses were between 18 and 35 years of age, whereas 21.4% of generalist nurses were between 36 and 55 years. 68.2% of specialist nurses and all generalist nurses were females. Most specialist nurses (77.3%) and all generalist nurses held a diploma in nursing and midwifery; 22.7% of specialists had a bachelor’s degree in nursing. All specialist nurses had received palliative care training, whereas 21.4% of generalist nurses reported having such training, including 14.3% who held a formal certification.

Most specialist nurses were in the early stages of their careers, with almost all having one to five years of experience. Generalist nurses displayed a broader range of practice experience, with 25% having more than 10 years of experience. Their clinical backgrounds spanned multiple inpatient and outpatient areas, most commonly oncology, while all specialist nurses were exclusively based in the palliative care unit.

**Table 3:** Sociodemographic and Professional Characteristics of FGD Participants (N = 50)

**Characteristics**	**Specialist nurses** **(n=22)**		**Generalist nurses** **(n=28)**	
	**Count**	**Percentage**	**Count**	**Percentage**
*Age in years*				
18–35	22	100.0	22	78.6
36–55	0	0.0	6	21.4
*Gender*				
Male	7	31.8	0	0.0
Female	15	68.2	28	100.0
*Professional qualification*				
Diploma in Nursing & Midwifery	17	77.3	28	100.0
Bachelor’s degree in Nursing	5	22.7	0	0.0
Certification in Palliative Care	0	0.0	4	14.3
Palliative care training (non-certification)	22	100.0	12	21.4
*Total Years of Experience*				
1–5 years	21	95.5	16	57.1
6–10 years	1	4.5	5	17.9
>10 years	0	0.0	7	25.0
*Area of Experience*				
Oncology (inpatient)	0	0.0	15	53.6
Emergency	0	0.0	2	7.1
Neurology (inpatient)	0	0.0	2	7.1
Medicine (inpatient)	0	0.0	3	10.7
Intensive Care Unit	0	0.0	3	10.7
Outpatient Department	0	0.0	3	10.7
Palliative care unit	22	100.0	0	0.0

Table 4 presents the sociodemographic and clinical characteristics of patients and caregivers. Most patients were aged above 65 years (45%), with a mean age of 60.8 years, and 55% were females. 40% had primary education, and 50% were homemakers. Oncology was the predominant specialty (50%), followed by cardiology (25%). Most patients (65%) reported disease duration of 1–5 years, and 55% resided in rural areas.

**Table 4:** Sociodemographic and Clinical Characteristics of Patients and Caregivers

**Characteristic**	**Patients (n=20)**		**Caregivers (n=15)**	
Part A: Sociodemographic Characteristics				
Age in years*	60.8 ± 13.7 (37–81)		43.5 ± 12.9 (25–63)	
	**Count**	**Percentage**	**Count**	**Percentage**
*Gender*				
Male	9	45	4	26.7
Female	11	55	11	73.3
*Educational Qualification*				
No formal education	1	5	0	0
Primary (Grades 1–5)	8	40	1	6.7
Middle (Grades 6–8)	3	15	4	26.6
Secondary (Grades 9–10)	4	20	1	6.7
Higher Secondary(Grades 11–12)	3	15	3	20
Graduate and above	1	5	6	40
*Occupation*				
Household	10	50	5	33.3
Agriculture	4	20	3	20
Laborers	1	5	1	6.7
Skilled workers	0	0	1	6.7
Service industry/Professionals	5	25	5	33.3
*Place of residence*				
Rural	11	55	11	73.3
Urban	9	45	4	26.7
Part B: Clinical Characteristics				
*Multimorbidity/Comorbidities*				
Yes	14	70	-	-
No	6	30	-	-
*Specialty of care*				
Nephrology	2	10	2	13.3
Oncology	10	50	4	26.7
Cardiology	5	25	1	6.7
Gastroenterology	2	10	2	13.3
Neurology	1	5	2	13.3
Pulmonology	0	0	4	26.7
*Duration of disease in years*				
< 1	6	30	3	20
1–5	13	65	8	53.3
6–10	1	5	4	26.7

*Data expressed as Mean ± Standard Deviation (Minimum-Maximum)

Caregivers were younger, with only 20% aged 55 years or older, and 73.3% were female. A higher proportion had graduate-level education (40%), and homemakers and service professionals each accounted for 33.3% of caregivers. Most caregivers were rural dwellers (73.3%). The patients they cared for were mostly in oncology (26.7%) and pulmonology (26.7%), followed by nephrology, gastroenterology, and neurology. The clinical conditions covered cardiology (congestive heart failure, cardiomyopathy), nephrology (end-stage renal disease), neurology (multiple sclerosis, Parkinson’s disease, stroke), gastroenterology (chronic liver disease), and pulmonology (chronic obstructive pulmonary disease). Caregivers reported that the duration of illness of their patients ranged primarily from 1 to 5 years (53.3%), with a smaller proportion having patients with illnesses longer than 6 years (26.7%).

### Emergent competency domains

Thematic analysis identified key competencies for palliative nursing in the Indian context, which were organized into eight domains reflecting empirical patterns and sociocultural realities: Foundations of Palliative Nursing Practice, Clinical Care: Symptom Management & Comfort, Communication & Interpersonal Relationship, Psychosocial, Cultural & Spiritual Support, Family & Caregiver Support, Collaboration & Care Coordination, Ethical, Legal & Professional Responsibilities.

Table 5: Domains, Themes, and Subthemes of Palliative Nursing Competencies. The detailed version with example codes is available as supplementary material (1). (Supplementary material 2)

**Table 5:** Domains, Themes, and Subthemes of Palliative Nursing Competencies

Domain	Theme	Subtheme
Foundations of Palliative Nursing Practice	Foundations of Palliative Nursing Practice	Holistic approach to PC Focus on quality of life Respect for patient choices
	Professional values and attitudes	Compassion and empathy Respect and dignity Non-judgmental care
	Knowledge and continuous learning	Understanding life-limiting illness Ongoing training and education Reflective practice
	Patient-centred approach	Advocacy for the patientIndividualized care
Clinical Care: Symptom Management & Comfort	Pain assessment and management	Pain & symptom assessment Non-pharmacological & pharmacological pain management Educating about narcotics & sedatives
	Symptom management	Symptom care in advanced illness Wound care & basic procedures Emergencies in PC Cultural aspects in symptom care
	Physical comfort and hygiene	Basic nursing procedures Complete personal care
	Ongoing symptom assessment	Continuous patient monitoring
Communication & Interpersonal Relationships	Need assessment and use of aids	Assess communication needs Use of communication aids Translator service
	Relationship Building	Encountering patients & families Handling difficult interactions
	Building trust and rapport	Spending time with the patient Provide realistic information Showing genuine care
	Active listening	Listening without interrupting Picking up unspoken worries
	Empathic communication	Speaking kindly Showing understanding Non-verbal skills
	Clear and honest information sharing	Giving correct information Avoiding medical jargon Dealing with collusion
	Managing difficult conversations	Handling anger or fear Mediating family conflict Maintain confidentiality
	Communicating with families	Updating family regularly Involving family in discussions Advocacy through communication
Psychosocial, Cultural & Spiritual Support	Person & Family Centred Care	Cultural humility Identify preferences & needs Provide dignity & safety Problem-solving & decisions
	Cultural & Spiritual Care	Cultural sensitivity at EOL Pain beliefs & practices Support spiritual needs Respond to spiritual distress
	Emotional support for patients	Providing comfort during fear
		Being present
	Addressing anxiety and depression	Recognizing emotional distress Linking to a counsellor/chaplain
	Family emotional support	Supporting family in grief& bereavement Helping family cope with stress
	Respect for cultural practices	Honouring rituals and customs Being sensitive to beliefs
	Spiritual care and support	Connecting with spiritual resources Facilitating last rites
Family & Caregiver Support	Emotional & Psychosocial Support	Breaking bad news Build trust & empathy Address emotional distress Coping & grief care Cultural aspects Listening to family worries
	Practical Support	Financial & resource help Mental health referral
		Mental health referral
	Education and guidance for the family	Teaching care tasks Family preparedness
	Encouraging family involvement	Involving and empowering them in daily care Helping family feel useful
	Facilitating family communication	Helping family talk openly Understanding family dynamics
	Linking to a counsellor or social worker	Connect to a counsellor Giving info about community help
	Caregiver self-care support	Reminding family to rest Encouraging respite
		Encouraging respite
Collaboration & Care Coordination	Teamwork & Referral	Multidisciplinary teamwork Working with other nurses Role clarity & boundaries Linking with the community
	Coordinating with other services	Referring to a specialist Linking with social workers
	Family involvement in the care plan	Including family in decisions Sharing updates regularly Cultural liaison
Ethical, Legal & Professional Responsibilities	Ethics & Advocacy	Legal & ethical practice Patient rights & autonomy Document advance care planning
	Self-Care & Professional Growth	Prevent burnout Build resilience Reflective practice Cultural humility
	Respecting patient rights and autonomy	Supporting patient choices Informed consent
	Maintaining confidentiality	Protecting patient information Discreet communication
	Advocacy for patient interests	Speaking up for vulnerable patients Reporting concerns
	Professional accountability	Following protocols Staying within the scope of practice
		Managing moral dilemmas
	Ethical decision-making & Legal awareness	Knowing the patient's legal rights Documentation for legal protection

### Foundations of palliative care

Participants emphasized that palliative nursing practice must be grounded in an understanding of palliative philosophy, illness trajectories, and holistic care addressing physical, emotional, social, and spiritual needs. Nurses were expected to have adequate knowledge of disease processes and anticipated outcomes to respond to patient and family concerns and build trust.

*“First*,* nurses should have good knowledge about our disease*,* treatment*,* and what will happen and everything. They should clear our doubts and win our trust.”* (IDI-P11).

As illness progressed, providing comprehensive, hands-on care became central, particularly for patients who were increasingly dependent.

*“We need to give them total care—pain medication*,* feeding*,* bathing… We should anticipate their needs and do what is necessary. Unlike others*,* these and their caregivers truly require a lot of psychosocial and spiritual support too.”* (FGD-7, P5).

Participants highlighted that the core goal of palliative care is comfort and quality of life, regardless of care setting. Specialist nurses emphasized that palliative principles should be integrated alongside curative treatment in acute and tertiary hospitals.

*“In India*,* patients requiring palliative care can be in any setting. So nurses in acute tertiary hospitals should also have this idea of care in addition to curative treatment.”* (FGD-5, P3).

Across stakeholder groups, non-discriminatory, respectful, and dignified care emerged as fundamental. Simple acts of compassion were viewed as essential to preserving dignity.

*“They are just treating me as their own grandfather. That is enough.”* (IDI-P5).

The fundamental qualities that support compassionate palliative nursing practice were found to be patience, empathy, and respect for patient autonomy.

*“We need a lot of patience and a kind heart to deal with these patients and their caregivers.”* (FGD-5, P2).

This domain reflects the basic knowledge, values, and holistic orientation required for palliative nursing practice.

### Clinical care: symptom management and comfort

Participants described physical comfort, symptom management, and end-of-life care as central to palliative nursing practice. Caregivers were often described as struggling to accept impending loss, frequently focusing on cure and perceiving care as insufficient.

*“Caregivers often struggle to accept the eventual loss of their patients… Most of them focus on a cure and appear to be unsatisfied with our care.”* (FGD-6, P3).

Controlling physical discomfort through comprehensive pain and symptom assessment, pharmacological and non-pharmacological interventions, and patient and family education was identified as a primary focus of care.

*“I cared for a patient who would scream and shout angrily… Once we started appropriate pain medication*,* she became calm and lived peacefully for another month.”* (FGD-6).

Participants emphasized that effective pain management requires skill in assessment, medication use, and interpreting patient behaviors that may reveal unexpressed suffering.

*“We are taught the use of various pain assessment scales and the WHO pain ladder.”* (FGD-5, P2).

Basic nursing care, including hygiene, elimination, and comfort, was described as essential for maintaining dignity and preventing complications, particularly for bedridden patients.

*“Hygiene and elimination needs are top priorities.”* (FGD-7).

Practical competencies such as wound care, management of non-healing wounds, pressure ulcers, tube feeding, tracheostomy and colostomy care, and maintaining skin integrity were considered core to ensuring comfort and quality of life.

*“Wound care is critical here… we even had patients with maggots in their wounds.”* (FGD-8).

Participants also emphasized the importance of competence in handling medical equipment and performing both invasive and non-invasive procedures, including the use of syringe pumps, oxygen concentrators, and non-invasive ventilation, to manage emergencies such as severe pain, breathlessness, hemorrhage, and seizures.

*“I never knew how to use syringe pumps*,* oxygen concentrators*,* and other non-invasive ventilations until recently.”* (FGD-7, P4).

Ensuring patient safety through adherence to protocols was emphasized, particularly when patients underestimated their limitations or engaged in risky behaviors.

*“Some insist on bathing alone… one patient even wanted to smoke in bed.”* (FGD-6).

End-of-life care was described as a sensitive and individualized aspect of practice, requiring timely recognition of deterioration and appropriate responses to ensure comfort and dignity.

*“Some patients deteriorate rapidly… whichever*,* we need to respond appropriately.”* (FGD-4, P3).

*“Making their death as peaceful and comfortable as possible is what really counts.”* (FGD-8, P1).

Overall, managing complex symptoms, responding to emergencies, attending to cultural beliefs, and prioritizing safety were viewed as fundamental responsibilities of palliative care nurse.

#### Communication and interpersonal relationships

Communication was identified as central to effective palliative care. Participants emphasized the importance of compassionate, honest, and respectful verbal and non-verbal communication in building trust and responding to individual patient and family needs.

*“We should feel they are approachable… without fear.”* (IDI-P15).

Meaningful nurse-patient relationships were thought to be characterized by approachability, attentive listening, and sincere concern. Nonverbal cues like touch reinforced emotional connection.

*“They really want to talk to you… That touch is there*,* you know.”* (IDI-P7; IDI-P15).

Addressing language barriers through interpreters and maintaining confidentiality during sensitive discussions, including those related to prognosis and end-of-life care, were identified as essential competencies. Participants highlighted the challenges of collusion, particularly when families requested that information be withheld from the patients.

*“Relatives mask the actual status and tell us not to communicate much with them.”* (FGD-8).

Fostering a no-blame attitude and observing nonverbal cues to spot discomfort or emotional distress were deemed crucial, particularly in emotionally charged situations.

*“If you can do something at this stage*,* you do. No need to blame.”* (IDI-P19).

Caregiver involvement was emphasized as integral to communication within the Indian context. Participants emphasized the importance of actively engaging caregivers in decision-making, respecting their presence, and supporting them as partners in care.

*“Instead of setting rules on us*,* they should allow us to be with our loved ones.”* (IDI-P18).

Facilitating family discussions, supporting shared decision-making, and promoting social connections were viewed as extended competencies that enhance continuity and quality of care.

#### Psychosocial, Cultural, and spiritual support

One of the key areas of palliative nursing competency that participants frequently highlighted was psychosocial, cultural, and spiritual support. To handle patients’ emotional sensitivity, it was believed that developing rapport and trust through empathetic communication was crucial.

*“I am very much worried.” (IDI-P15)*.

A non-discriminatory and nonjudgmental approach, grounded in awareness of patients’ social, cultural, and economic contexts, was described as central to meaningful care.

*“We are poor people from a village… Do not know all these big things.” (IDI-P17)*.

Simple acts of presence, such as sitting with patients or taking them outside, were perceived as powerful in reducing isolation, promoting comfort, and facilitating emotional expression.

*“At least I feel I am part of this world when these are done.” (IDI-P3)*.

*Such interactions also enabled patients to share concerns*,* articulate preferences*,* and gradually prepare for the end of life.*

*“During those times*,* they share their feelings… and what they would like for their final journey.” (FGD-8*,* P1)*.

*Participants highlighted the importance of recognizing psychological distress*,* including fear*,* anxiety*,* and depression*,* and involving counselors or other mental health resources when needed.*

*“We notice fear or emotional issues and involve counsellors to help them.” (FGD-8)*.

Understanding differences in patients’ educational, socioeconomic, and cultural backgrounds was considered necessary for delivering acceptable and effective care.

*“Many poor village patients accept palliative care more easily.” (FGD-5*,* P4)*.

Spiritual and cultural care, particularly at the end of life, was described as integral to maintaining dignity. Facilitating religious practices, respecting beliefs, and supporting culturally appropriate death and mourning rituals were viewed as essential nursing responsibilities.

*“Different religions have different ways. We have to follow their request to handle the body.” (FGD-6*,* P3)*.

Participants also emphasized supporting families before, during, and after death by preparing them for what to expect and guiding them through bereavement.

*“We never knew what to do once he was gone.” (IDI-P12)*.

Overall, alleviating fear, fostering emotional safety, and providing culturally and spiritually responsive care were described as central to ensuring a dignified and compassionate end-of-life experience.

*“If people are kind*,* that’s enough… they can help me be without fear.” (IDI-P1)*.

#### Family and caregiver support

Participants emphasized family and caregiver support as an integral component of palliative care. Understanding and respecting family dynamics were considered essential for providing inclusive and responsive care. Educating families and offering practical guidance on caregiving responsibilities helped build confidence and caregiving capacity.

*“The nurses… treated me as family. I never felt alone.”* (IDI-19).

Participants also emphasized the importance of connecting families with relevant services and available resources, including government welfare schemes.

*“Some schemes are available for our treatment from the government. They should tell us how to get them.”* (IDI-7).

Recognizing caregiver burden and providing appropriate support, such as counselling or respite, were considered crucial for sustaining caregiver well-being. Caregivers often experienced emotional distress related to anticipatory grief and competing life stressors.

*“I felt anger*,* sadness*,* frustration… I just needed someone to ask how I was feeling.”* (IDI-P5).

Participants noted that sensitizing caregivers to the reality of the patient’s condition, particularly when caregivers were in denial, was challenging and required careful, paced communication.

*“We can never predict the caregivers’ reaction when they learn there is no hope.”* (FGD-5, P3).

Identifying caregivers at risk of complicated grief and escalating concerns through timely referral to counsellors or senior staff were described as essential aspects of intuitive and professional nursing care.

*“If we find that some relatives are not coping*,* we inform the counsellors for follow-up and support.”* (FGD-7, P1).

#### Multidisciplinary collaboration and care coordination

Effective collaboration within multidisciplinary teams was identified as essential for delivering coordinated and holistic palliative care. Participants emphasized the importance of collaborating closely with physicians, nurses, social workers, counselors, and other healthcare professionals to tailor care to patients’ preferences and needs.

*“If nurses can tell doctors about us after they saw us last*,* it will be a great use for us.”* (IDI-P12).

Nurses’ roles as liaisons between patients, families, and the healthcare team were viewed as central to continuity of care. Timely referrals to appropriate services, including counseling, home care, hospice, and financial support, were considered critical in addressing complex patient and family needs.

*“We usually find out what is bothering the patients and then get the counsellors involved.”* (FGD-7, P2).

Integrated decision-making was facilitated by active involvement in case discussions, interdisciplinary care planning, and collaboration with allied health professionals. Nurses also emphasized the importance of accurate and timely documentation to deliver safe, informed, and consistent care across various care settings.

*“Documenting discussions and care is one of our important tasks.”* (FGD-7, P3).

#### Ethical, Legal, and professional responsibilities

Participants described ethical and legal competence as central to the delivery of quality palliative care. Respect for patient autonomy and support for informed decision-making were viewed as core professional responsibilities. Nurses were often involved in facilitating informed consent as care progressed toward the end of life, emphasizing the need for adequate explanation and sufficient time during the consent process.

*“Taking consent for many things*,* people just ask us to sign. They should take time and explain.” (IDI-19)*.

Proper documentation of advance care planning, including advance directives and Do-Not-Resuscitate (DNR) orders, was considered essential to honoring patient preferences and ensuring legal clarity. Participants noted that incomplete or inaccurate documentation could lead to ethical and legal challenges, particularly during invasive procedures and end-of-life decision-making.

*“We must always obtain signatures on consent forms and file them properly… especially for DNR orders.” (FGD-2*,* P5)*.

Participants also highlighted their responsibility in ensuring that post-death procedures were conducted in accordance with legal requirements, including appropriate handover of the body and completion of necessary documentation.

*“We must ensure that the body is handed over to the appropriate person and that all legal formalities are completed.” (FGD-6*,* P3)*.

Ethical dilemmas were commonly encountered in palliative care, particularly when patient wishes, family expectations, and clinical realities differed. In such situations, participants emphasized advocacy for patient rights and the importance of team discussion and ethical consultation.

The emotional demands of end-of-life care underscored the importance of nurse self-care and professional resilience. Participants described the need to manage their own emotions while providing emotional support to patients and families.

*“We shouldn’t cry in front of patients; need to stay strong*,* comfort them*,* and help them adjust.” (FGD-6*,* P5)*.

Recognizing personal emotional distress and seeking support were viewed as necessary for maintaining emotional balance.

*“When everyone around is crying*,* we also feel like crying.” (FGD-5*,* P2)*.

Reflective practice and debriefing were described as helpful in processing difficult experiences and sustaining professional well-being. Ongoing education and training were considered essential for maintaining competence, particularly given the limited coverage of palliative care in formal nursing education.

*“Now we know this is a wide area.” (FGD-5*,* P1)*.

Participants also emphasized the importance of aligning practice with institutional policies and national legal frameworks to ensure consistency, accountability, and the delivery of safe care.

*“We should know about the legal side… Our institution has one document.” (FGD-3*,* P1)*.

Together, these domains and subthemes reflect a comprehensive, contextually relevant competency framework for palliative nursing practice. They highlight not only technical and clinical skills but also the values, attitudes, cultural humility, and interpersonal skills that enable nurses to deliver truly holistic, patient- and family-centered care.

## Discussion

This study explored the competencies required for nurses to deliver palliative care in the Indian context, drawing on the perspectives of nurses, patients, and caregivers. The findings reveal that palliative nursing competence is holistic and integrative, encompassing not only clinical and technical skills but also value-based practice, communication, cultural sensitivity, family engagement, ethical accountability, and professional self-sustainability [34]. Rather than functioning as isolated domains, these competencies were carried out simultaneously in everyday clinical practice, shaped by contextual realities, resource constraints, and sociocultural norms [33]. By articulating competencies across different levels of palliative care practice, this study addresses role-specific expectations for palliative nursing in the Indian context.

### Influence of patient and caregiver characteristics on palliative nursing competencies

Patient and caregiver characteristics provided critical contextual grounding for the competencies identified. The increased prevalence of people living with chronic, progressive illnesses calls for the expanding scope of palliative care beyond terminal oncology to cover long-term symptom management, psychosocial support, and sustained care across extended illness trajectories [35]. This shift helps explain participants’ emphasis on anticipatory care, continuity, and long-term emotional engagement as core nursing competencies. In this regard, the findings extend existing literature by illustrating how palliative nursing competencies must adapt to prolonged care rather than episodic end-of-life encounters [36]. While prior studies, largely from high-income settings, have focused on specialist services, this study highlights the sustained relational work required of nurses caring for patients with chronic, progressive illnesses over extended periods in the Indian context.

The high proportion of patients from rural backgrounds and with limited formal education further stresses the centrality of communication, trust-building, and caregiver education within palliative nursing practice. In contexts of low health literacy, nurses function as interpreters of medical information and facilitators of shared decision-making, positioning communication as a core professional competency. This conclusion builds on previous research by demonstrating how communication skills in palliative care are linked to broader issues of equity, access, and social vulnerability [37]. Participants in this study described communication as an ongoing process of translation, reassurance, and negotiation rooted in social and cultural norms, in contrast to studies carried out in environments with higher health literacy and access to specialized services.

The significant participation of family caregivers, mostly women, highlights the value of family-centered care and reflects the deeply ingrained sociocultural caregiving traditions in India. Competencies related to caregiver assessment, emotional support, and bereavement preparation emerged as integral to nursing practice, highlighting the extent to which patient outcomes are intertwined with caregiver well-being [38]. Collectively, these findings illustrate how patient and caregiver profiles actively shape the implementation of palliative nursing competencies and reinforce the necessity of culturally and socially responsive care models.

### Application of palliative nursing competencies in practice

Participants emphasized that a holistic approach to palliative care goes beyond symptom control to include psychosocial, emotional, and spiritual support [39]. In line with the fundamental values of dignity, autonomy, and quality of life, compassion and respect were described as essential to care delivery [40]. The ability to anticipate patient and family needs, communicate effectively across the illness trajectory, and respond empathetically to distress was viewed as essential for building trust and sustaining therapeutic engagement, consistent with existing evidence [33].

While these competencies were shared across care settings, participants distinguished between advanced competencies expected of specialist palliative care nurses, such as management of complex symptoms, ethical decision making and interdisciplinary leadership, and the foundational competencies required of generalist nurses, such as basic symptom management, communication, and family support [41].

Ethical and legal competency emerged as essential components of practice. Continuous education was seen as necessary for strengthening ethical decision-making and enabling nurses to handle complex end-of-life situations while safeguarding patient rights [34, 42]. Nurses’ roles in advocacy, documentation of care preferences, and adherence to professional standards also highlight the importance of multidisciplinary collaboration and coordination across care settings to support continuity and coherence of care [43].

Cultural and spiritual responsiveness was integral to practice, particularly in multicultural palliative care contexts [44]. Participants emphasized cultural humility, spiritual support, and respect for rituals and beliefs, especially at the end of life, as essential to delivering person-centered care [45, 46]. Nurses also provided education, emotional support, and anticipatory guidance to address caregiver burden and uncertainty, consistent with a family-centered model of palliative care [47].

The emotional demands of palliative care nursing require the need for self-care, reflective practice, and peer support in maintaining professional well-being. Participants identified resilience-building techniques as essential for reducing compassion fatigue and burnout, reinforcing concerns regarding workforce sustainability in palliative care [48, 49]. Finally, FGD participants identified competencies related to informatics and accurate documentation as highly essential for ensuring patient safety, continuity of care, and legal protection within the evolving healthcare systems [47].

### Implications for nursing education and clinical training

The findings have clear implications for nursing education and workforce development, highlighting the need for structured, competency-based training across all levels of practice [50]. Owing to the extensive need for palliative care across the illness continuum, all nurses should be trained to provide competent palliative care. Integrating palliative care principles into undergraduate curricula and early-career training may enhance nurses’ preparedness to deliver quality palliative care across diverse clinical settings [51]. The results indicate that structured orientation programs emphasizing family communication, basic symptom management, and culturally competent end-of-life care will be beneficial for nurses in various healthcare settings. The need for improving education on ethical issues, legal obligations, and relevant national rules is drawn from the participants’ emphasis on documentation, informed consent, advocacy, and advance care planning. Clinical judgment and advanced competency development may be especially well-supported by educational strategies such as case discussions, mentorship, simulation-based learning, and reflective practice [52]. Given the emotional intensity of palliative care practice, incorporating training on emotional regulation, reflective practice, peer support, and burnout prevention into pre-service education and ongoing in-service programs is critical. Such initiatives are necessary not only for sustaining the palliative nursing workforce but also for maintaining the quality and compassionate care over time [53].

### Strengths

This study draws on a diverse participant pool, including generalist and specialist nurses from multiple specialties, as well as patients and caregivers from various linguistic and cultural backgrounds in the Indian subcontinent. This enhances the generalizability/transferability of the findings to broader palliative care contexts in India and similar settings. Using both FGDs and IDIs enabled data triangulation and a more refined understanding of the competencies needed in PC from both provider and recipient perspectives. Thematic analysis was conducted via the rigorous Braun and Clarke framework, ensuring a systematic and transparent approach to theme development. The competency statements were directly grounded in participant narratives, providing contextual richness and real-world relevance to the identified domains and subdomains.

### Limitations

Although the study included participants speaking at least five different regional languages, language translation and interpretation may have led to a subtle loss of meaning in some narratives. The study’s findings are based on participants from tertiary care and specialized palliative care centres, which may not fully capture the competencies needed in other settings. Participant selection through purposive sampling may have introduced selection bias. The presence and style of facilitators and translators may also have influenced the depth and direction of the discussions. Additionally, the cultural context in which the study was conducted, particularly the beliefs and norms surrounding illness, death, and caregiving in India, may limit the transferability of the findings to other contexts. As the data reflect perceptions at a single point in time, they may not capture evolving competencies or shifts in palliative care practice and policy.

## Conclusion

The qualitative analysis of FGDs with nurses and IDIs with patients and caregivers has identified culturally and contextually specific nursing competencies that are essential for the effective delivery of palliative care. There is a significant overlap between the expectations expressed by patients and caregivers and the qualities that nurses believe are essential. These findings provide a crucial foundation for developing a context-relevant palliative nursing competency framework. Upon validation through rigorous methodologies, such a framework may serve as a valuable resource for nursing education, clinical practice, healthcare management, and future research in palliative care.

## Supplementary Information

Supplementary Material 1.

Supplementary Material 2.

## Data Availability

The data supporting these study findings are available from the corresponding author upon reasonable request.

## Abbreviations

PC: Palliative Care
FGD: Focus Group Discussion
IDI: In Depth Interview
COREQ: Consolidated Criteria for Reporting Qualitative Research
