# Supplementary material for: Exploring stakeholder perspectives on nursing competencies in palliative care in India: a qualitative inquiry
Source: BMC Palliat Care. 2026 Feb 5;25:60. doi: 10.1186/s12904-026-01998-1 (PMC12964646; doi:10.1186/s12904-026-01998-1)
Supplement: Supplementary file 2 — Supplementary Material 2. [file 12904_2026_1998_MOESM2_ESM.docx]

| Domains, Themes, Subthemes and example codes of Palliative Nursing Competencies | | | |
| --- | --- | --- | --- |
| **Domain** | **Theme** | **Subtheme** | **Example Codes** |
| \| **Foundations of Palliative Nursing Practice** \| \| --- \| | \| Palliative care philosophy and principles \| \| --- \| | Holistic approach to PC | *“mind, body, family all matter”, “not just treating disease” (FGD- 5,P2)* |
|  |  | Focus on quality of life | *“make them comfortable, maybe no cure anymore.”(FGD- 3 P4)* |
|  |  | Respect for patient choices | *“what we want- not what they like.” (IDI- 8)* |
|  | \|  \| \| --- \|  \| Professional values and attitudes \| \| --- \| | Compassion and empathy | *“be kind, that’s the heart of this work.”(FGD- 5, P4)* |
|  |  | Respect and dignity | *“give us some respect as human beings.” (IDI- 22)* |
|  |  | Non-judgmental care | *“never judge their choices or beliefs.” (FGD-6, P3)* |
|  | Knowledge and continuous learning | Understanding life-limiting illness | *“know disease stages”, “prepare family for what’s next” (FGD 6, P3)* |
|  |  | Ongoing training and education | *“to handle new equipment” ( FGD-7, P5) , “medication titration” (FGD-5, P1)* |
|  |  | Reflective practice | *“After every case, I think about what I could do better.” (FGD- 6, P2)* |
|  | Patient-centered approach | Advocacy for the patient | *“have to speak for your patient” (FGD- 3, P2)* |
|  |  | \|  \| \| --- \|  \| Individualized care \| \| --- \| \|  \| | *“each patient is unique” (FGD- 3, P4)* |
| **Clinical Care: Symptom Management & Comfort** | Pain assessment and management | Pain & symptom assessment | *“ask about pain in simple words”, “watch face for pain signs” (FGD-2, P1)* |
|  |  | Non-pharmacological & pharmacological pain management | *“use massage if needed”, “give pain meds on time” (FGD5, P1)* |
|  |  | Educating about narcotics & sedatives | *“Explain about pain medications.” “dependency on pain meds” (FGD-6, P3)* |
|  | Symptom management | Symptom care in advanced illness | *“manage breathlessness”, “comfort when dying” (FGD-1, P1)* |
|  |  | Wound care & basic procedures | *“do dressing gently(IDI-17), “keep wounds clean” (IDI-23), “dressing wounds with maggots” (FGD- 7, P4)* |
|  |  | Emergencies in PC | *“act fast in sudden pain” (FGD-6, P4), “yelling with pain in the middle of the night” (FGD-6, P2) “ “ bleeding too much”(FGD-5, P3)* |
|  |  | Cultural aspects in symptom care | *“respect home remedies”, “know local healing beliefs.”* |
|  | Physical comfort and hygiene | Basic nursing procedures | *“Catheterization, Canulation, Nasogastric tube insertion, PEG feeding” (FGD- 1, P1)* |
|  |  | Complete personal care | *“A–Z care from feeding, bathing, and providing comfort.” (FGD-6, P3)* |
|  | Ongoing symptom assessment | Continuous patient monitoring | *“know what is going on with us” (IDI-1)* |
| **Communication & Interpersonal Relationship** | Need assessment and use of aids | Assess communication needs | *“see if they understand”, “check for any barrier” (FGD5, P2)* |
|  |  | Use of communication aids | *“show pictures if needed”, “write down if can’t talk” (FGD-6P3)* |
|  |  | Translator service | *“ get translator help when required” (FGD- 2, P5)* |
|  | Relationship Building | Encountering patients & families | *“listen fully” (IDI-7), “spend time with them” (FGD-3, P3)* |
|  |  | Handling difficult interactions | *“stay calm with angry family” (FGD-4, P4), “negotiate with relatives” (FGD-4, P3)* |
|  | Building trust and rapport | Spending time with patient | *“Only work, no talking.”(IDI-5)* |
|  |  | Provide realistic information | *“never give false promise, No point giving fake hope” (FGD-2, P1),* |
|  |  | Showing genuine care | *“Genuine people do from the heart” (IDI-23)* |
|  | Active listening | Listening without interrupting | *“Sometimes they just want to talk, we listen.”(FGD-7, P2)* |
|  |  | Picking up unspoken worries | *“You can tell by their face when something’s wrong.” (FGD-5-P3)* |
|  | Empathic communication | Speaking kindly | *“be kind, not do this, do that” (IDI-24)* |
|  |  | Showing understanding | *“Your problem- not our worry” (IDI-26)* |
|  |  | Non-verbal skills | *“That touch is enough, you know” (IDI- 6)* |
|  | Clear and honest information sharing | Giving correct information | *“Tell them what is really happening.” (FGD-6, P3)* |
|  |  | Avoiding medical jargon | *“simple words, no medical words” (FGD- 4, P1)* |
|  |  | Dealing with collusion | *“Families keep diagnosis secret” (FGD-3, P4). “Patients don’t know what they have.” “not to reveal any information to patients” (FGD-6, P3)* |
|  | Managing difficult conversations | Handling anger or fear | *“Families get upset”, “shout at the nurses' station” (FGD-3, P5)* |
|  |  | Mediating family conflict | *“Sometimes family members fight, we help them talk.” (FGD-2, P3)* |
|  |  | Maintain confidentiality | *“keep secrets safe”, “don’t share without consent”, “what I tell the nurse should be with her- not tell my son” (IDI-27)* |
|  | Communicating with families | Updating family regularly | *“We explain changes to the family.” (FGD-2, P5)* |
|  |  | Involving family in discussions | *“Include them in decisions too.” (FGD-3, P2)* |
|  |  | Advocacy through communication | *“Ask us to speak to doctors” (FGD-4-P3)* |
| **Psychosocial, Cultural & Spiritual Support** | Person & Family Centered Care | Cultural humility | *“learn from patients’ beliefs”, “respect family ways” (FGD-5, P5)* |
|  |  | Identify preferences & needs | *“ask what they want” (FGD-5, P2), “respect customs” (FGD-3, P2)* |
|  |  | Provide dignity & safety | *“keep them clean and covered, give privacy” (FGD-2, P1)* |
|  |  | Problem-solving & decisions | *“help decide what’s best” (IDI-3), “tell us what to do”(IDI-17)* |
|  | Cultural & Spiritual Care | Cultural sensitivity at EOL | *“know rituals for last moments”, “help prayers at bedside” (FGD-2, P5)* |
|  |  | Pain beliefs & practices | *“some think pain is fate” (FGD-6, P3)* |
|  |  | Support spiritual needs | *“ask if they want priest”, “listen to spiritual worries” ( FGD-4, P3&1)* |
|  |  | Respond to spiritual distress | *“sit with them when scared” (FGD-3,P1), “wanted to do pooja” (FGD-4- P3)* |
|  | Emotional support for patients | Providing comfort during fear | *“hold their hand when they’re scared.” “scared of floating off”(FGD-5, P6)* |
|  |  | Being present | *“Sometimes just sitting quietly helps” (FGD- 3, P4)* |
|  | Addressing anxiety and depression | Recognizing emotional distress | *“how is life going to be?” (IDI-29). “I have no more tears” (IDI-16)* |
|  |  | Linking to a counselor/chaplain | *“If they cry a lot, we call a counselor” (FGD-6, P6)* |
|  | Family emotional support | Supporting family in grief& bereavement | *“Talk to families when they feel hopeless.” (FGD- 5, P4)* |
|  |  | Helping family cope with stress | *“Nobody even asked me what I was going through.” (IDI- 5)* |
|  | Respect for cultural practices | Honoring rituals and customs | *“We allow family to do prayers.” (FGD1, P4)* |
|  |  | Being sensitive to beliefs | *“cannot eat before bath” (IDI- 27), “no onion in my food” (IDI- 22)* |
|  | Spiritual care and support | Connecting with spiritual resources | *“poojari, priest or imam if they want.” (FGD- 3, P4)* |
|  |  | Facilitating last rites | *“We help arrange prayers when they pass.” (FGD-1, P4)* |
| **Family & Caregiver Support** | Emotional & Psychosocial Support | Breaking bad news | *“tell truth step by step”, “give time to process” (FGD- 5, P6)* |
|  |  | Build trust & empathy | *“listen without judging” (FGD7, P1), “cancer is due to chewing betel leaves they said.” (IDI_29)* |
|  |  | Address emotional distress | *“watch for hidden tears”, “ask about stress” (FGD- 6, P5)* |
|  |  | Coping & grief care | *“support in grief”, “stay in touch after death” (FGD-7, P,4&3)* |
|  |  | Cultural aspects | *“know family mourning ways”, “support traditional rites.”* |
|  |  | Listening to family worries | *“Sometimes family just wants to vent — we listen.” (FGD- 6, P2) “ “Sisters (nurses) eventually became like second family for me.” (IDI-30)* |
|  | Practical Support | Financial & resource help | *“getting Ayushman Bharat” (IDI-7)* |
|  |  | Mental health referral | *“refer if signs of depression, link to a counselor” (FGD-5, P1)* |
|  | Education and guidance for family | Teaching care tasks | *“how to change position or clean wounds.” (FGD-4, P2)* |
|  |  | Family preparedness | *“We tell them what signs to look for as condition changes.” (FGD-5, P4)* |
|  | Encouraging family involvement | Involving and empowering them in daily care | *“Let them feed or massage the patient if they want.” (FGD-5, P4) “Now I know almost everything to care for my father at home.” (IDI-13)* |
|  |  | Helping family feel useful | *“helps to cope when they feel they are doing something.” (FGD- 4. P3)* |
|  | Facilitating family communication | Helping family talk openly | *“Doctors and nurses should find out from us (caregiver)” (IDI-9)* |
|  |  | Understanding family dynamics | *“Sometimes patients and their caregivers don’t get along.” (FGD4, P6)* |
|  | Linking to a counselor or social worker | Connect to counsellor. | *“Identify and refer to a counsellor” (FGD6, P1)* |
|  |  | Giving info about community help | *“take the patient and go… but where?.” (IDI- 24)* |
|  | Caregiver self-care support | Reminding family to rest | *“Used to ask me to go home and sleep for a while(caregiver)”. (IDI-24)* |
|  |  | Encouraging respite | *“Sometimes we suggest someone else sits in for a while.” (FGD- 6,P3)* |
| **Collaboration & Care Coordination** | Teamwork & Referral | Multidisciplinary teamwork | *“work with doctors, physiotherapists , social worker, counsellors etc”, “plan as team” (FGD-5, P4)* |
|  |  | Working with other nurses | *“handing over the shift” (FGD-3, P1)“new shift nurses will not know about us.” (IDI-10)* |
|  |  | Role clarity & boundaries | *“know my limits”, “don’t overstep” (FGD- 4, P2)* |
|  |  | Linking with the community | *“nobody told about palliative center”(IDI-11) “Palliative visits from government centers” (IDI-13)* |
|  | Coordinating with other services | Referring to a specialist | *“follow up referrals, “facilitate referrals.” (FGD-2, P1)* |
|  |  | Linking with social workers | *“Sometimes family needs financial help, we call a social worker.” (FGD-7, P5)* |
|  | Family involvement in care plan | Including family in decisions | *“make us go out during rounds, but we should know(caregiver)” ( IDI- 13)* |
|  |  | Sharing updates regularly | *“Inform family of changes, even call and inform”(FGD- 7, P5), “Daughter is calling nurses” (IDI-14)* |
|  |  | Cultural liaison | *“respect family traditions” (FGD-4, P1)* |
| **Ethical, Legal & Professional Responsibilities** | Ethics & Advocacy | Legal & ethical practice | *“follow DNR order” (FGD-3, P5) “respect patient choice” ( FGD-6, P2)* |
|  |  | Patient rights & autonomy | *“allow us to decide”(IDI-4), “facilitate communication with doctors for decision making” (FGD-3, P4)* |
|  |  | Document advance care planning | *“write down wishes”(FGD-6, P5), “record DNR clearly” (FGD-3, P2)* |
|  | Self-Care & Professional Growth | Prevent burnout | *“take breaks to stay fresh”, “talk to peers” (FGD-5, P3)* |
|  |  | Build resilience | *“bounce back from sad days” (FGD-3, P2), “stay strong in loss” (FGD-7, P3)* |
|  |  | Reflective practice | *“learn from my cases, think back on what I did” (FGD-5, P2)* |
|  |  | Cultural humility | *“never judge beliefs” (FGD-3, P2), “accept different customs” (FGD5, P4)* |
|  | Respecting patient rights and autonomy | Supporting patient choices | *“they have the right to refuse or even change facilities.” ( FGD4, P3)* |
|  |  | Informed consent | *“tell them everything before getting a signature” (IDI-11)* |
|  | Maintaining confidentiality | Protecting patient information | *“We don’t talk about them outside.” (FGD5, _4)* |
|  |  | Discreet communication | *“discuss sensitive things in front of everyone.” (FGD- 5, P6)* |
|  | Advocacy for patient interests | Speaking up for vulnerable patients | *“family imposes on patients” (FGD3, P3)* |
|  |  | Reporting concerns | *“report abuse if noticed” (FGD-4, P5)* |
|  | Professional accountability | Following protocols | *“follow PC rules”, “work as per national guide”, “EOLC protocols.” (FGD-6, P5)* |
|  |  | Staying within the scope of practice | *“ if not within my scope, just get the right person to do,” (FGD- 6, P3)* |
|  | \|  \| \| --- \|  \| Ethical decision-making \| \| --- \| | Managing moral dilemmas | *“when family wants everything done, but patient suffers.” (FGD-2, P5)* |
|  | Legal awareness | Knowing the patient's legal rights | *“Patient rights, living will” (FGD-4, p3)* |
|  |  | Documentation for legal protection | *“important to write down everything we do.” (FGD1, P3)* |
